# CCL21/CCR7 Axis Contributes to Trophoblastic Cell Migration and Invasion in Preeclampsia by Affecting the Epithelial Mesenchymal Transition via the ERK1/2 Signaling Pathway

**DOI:** 10.3390/biology12020150

**Published:** 2023-01-18

**Authors:** Zheng Liu, Jie He, Pingsong Jin, Yuxin Ran, Nanlin Yin, Hongbo Qi

**Affiliations:** 1 Department of Obstetrics, The First Affiliated Hospital of Chongqing Medical University, Chongqing 400016, China; 2 Chongqing Key Laboratory of Maternal and Fetal Medicine, Chongqing Medical University, Chongqing 400016, China; 3 Department of Obstetrics, Women and Children’s Hospital of Chongqing Medical University, Chongqing 401147, China; 4 Department of Center for Reproductive Medicine, The First Affiliated Hospital of Chongqing Medical University, Chongqing 400016, China

**Keywords:** CCL21/CCR7, preeclampsia, EMT, ERK1/2 pathway, trophoblast

## Abstract

**Simple Summary:**

Preeclampsia is a severe complication of human pregnancy. Despite intensive research efforts, effective treatment is still lacking. Impaired extravillous trophoblast invasion and incomplete spiral artery remodeling are thought to be the initial factors of PE. Furthermore, CCL21 has been widely reported to be associated with the metastasis of cancer cells. Considering the similarities between trophoblasts and cancer cells, we speculated that CCL21 might also affect trophoblast invasion and migration. In this study, we found the expression level of CCL21 was significantly lower in the PE group. In vitro study showed that CCL21 promoted trophoblast cell EMT processes and further enhanced its migration and invasion ability through the ERK1/2 signaling pathway. A mouse model of PE was established with L-NAME, and we obtained similar results. These data suggest that the CCL21/CCR7 axis affects EMT by activating the ERK1/2 signaling pathway, thereby affecting the migration and invasion of trophoblast cells. This contributes to effective therapeutic strategies for PE.

**Abstract:**

Preeclampsia (PE) is a pregnancy-related disorder that is a leading cause of maternal death. The failure of spiral artery remodeling due to insufficient trophoblast migration and invasion is critical in the pathogenesis of PE. Recently, the CC motif chemokine ligand 21 (CCL21) has been widely linked to cancer cell invasion and migration. However, their potential mechanisms are still unknown. In this study, we found that CCL21 expression was significantly lower in the PE group than that in the control group. In vitro experiments revealed that recombinant CCL21 could promote trophoblast cell epithelial-to-mesenchymal transitions (EMTs) and improve migration and invasion. Furthermore, an inhibitor of the ERK1/2 signaling pathway inhibited the CCL21-induced EMT process. Finally, a PE mouse model was established using the NOS inhibitor L-NAME, and we obtained similar results, with downregulated CCL21 and EMT biomarkers and upregulated CCR7. Taken together, these findings suggest that the CCL21/CCR7 axis influences EMT by activating the ERK1/2 signaling pathway, thereby affecting trophoblast cell migration and invasion, which may play a crucial role in the pathogenesis of PE.

## 1. Introduction

Preeclampsia (PE), a specific pregnancy disorder, is characterized by new-onset hypertension after 20 weeks of gestation. It affects approximately 5% to 8% of pregnancies globally each year [1]. Furthermore, PE is associated with an increased risk of preterm birth and fetal growth restriction, both of which pose serious risks to the mother and fetus [2]. Currently, the only effective treatment for PE is to terminate the pregnancy. Despite unrelenting efforts over the last decade to investigate the pathology of PE, its pathogenesis is still not fully elucidated. Several mechanisms have been proposed so far, including immune inflammatory imbalance, genetic and epigenetic imprinting, uteroplacental ischemia, and impaired trophoblast invasion [3,4,5]. Among the mechanisms mentioned above, insufficient trophoblast invasion is important in the pathogenesis of PE.

Normal placental implantation is necessary for a successful pregnancy. Uterine spiral arteries are converted into high-capacity, high-flow channels to supply the developing embryo with the necessary nutrition [6]. Therefore, the cytotrophoblasts’ development into extravillous trophoblasts (EVTs) is crucial for transforming the arteries into high-volume conduits. Cytotrophoblasts fail to transit from the proliferative epithelial subtype to the invasive endothelial subtype in placentas, which results in the inadequate remodeling of the spiral arteries that are going to develop preeclampsia [7]. It has been demonstrated that the developmental epithelial-to-mesenchymal (EMT) transition and the process of cytotrophoblasts differentiating into EVT are similar [8]. Epithelial cells can become mesenchymal cells with mesenchymal cell appearance and features via a physiological process called EMT [9]. The loss of epithelial markers, such as E-cadherin, and the increase in mesenchymal markers, such as N-cadherin and vimentin, are the hallmarks of EMT [10,11]. During normal placentation, the EVTs lose their ordered epithelial phenotype and transform into a migratory and invasive mesenchymal phenotype, allowing them to infiltrate the mother’s decidual stroma and blood vessels [12]. However, the deregulation of EMT impairs the trophoblasts’ capacity to migrate and invade effectively. These results highlight the significance of controlling EMT throughout the trophoblast’s cell growth.

At the maternal fetal interface, a variety of cytokines, chemokines, hormones, and growth factors control the biological processes of trophoblast cells in an autocrine and paracrine manner. Trophoblast adhesion, migration, and invasion are impacted by these variables [13]. Low molecular weight peptides known as chemokines have a variety of functions in controlling how cells operate biologically. The G protein-coupled receptor (GPCR) family of surface chemokine receptors is how chemokines carry out their biological function [14]. As a seven-fold transmembrane GPCR, chemokine receptor 7 (CCR7) is expressed by a variety of tumor types, including breast cancer, gastric carcinoma, and colorectal cancer [15]. Indeed, CCR7 is a receptor for the C-C chemokine ligand (CCL21), which is a significant member of the CCL family. It has been shown that the interaction between CCL21 and CCR7 facilitated the growth, invasion, and metastasis of tumors. For instance, by influencing the EMT pathway, CCL21/CCR7 facilitated the spread of pancreatic cancer cells [16]. Another study found that the link between CCL21 and CCR7 enhanced oral squamous cell carcinoma EMT [17]. The promotion of cancer cell migration into micro lymphatic capillaries by CCL21/CCR7 has also been observed in breast cancer [18] and esophageal squamous cell carcinoma [19]. Recent research suggested that common pathways between trophoblast and cancer cells may have co-evolved. As a result, we hypothesize that CCL21/CCR7 may engage in the pathophysiological mechanism of PE by controlling EMT to alter trophoblasts’ biological processes. The current study aims to explore the expression pattern of CCL21/CCR7 in the placenta of normal and PE pregnancies and to further analyze the molecular mechanism of CCL21/CCR7 participation in the development of PE.

## 2. Materials and Methods

### 2.1. Obtaining Clinical Samples

The First Affiliated Hospital of Chongqing Medical University served as the source for all subject recruitments. The American College of Obstetrics and Gynecology’s clinical recommendations served as the foundation for the PE diagnosis. Major pregnancy problems, such as gestational diabetes mellitus (GDM), chronic hypertension illnesses, viral diseases, and autoimmune diseases, were all excluded from the study. After elective cesarean sections were performed on patients with PE (*n* = 20) and normotensive pregnancies (*n* = 20), decidual and placental tissues were obtained. Table S1 listed the clinical characteristics of the participants that were enrolled. The bloodstain on tissues was removed from all samples by washing them several times in sterile phosphate-buffered saline (PBS). The samples were cleaned and then kept in refrigerators at −80 °C until testing.

### 2.2. Animal Model

The eight-week-old CD1 mice were purchased the Hunan SJA Laboratory Animal CO., LTD, and they were kept in a controlled setting with a 12 h light/12 h dark cycle. All mice had unrestricted access to normal food and water. The Institutional Animal Care and Use Committee of the First Affiliated Hospital of Chongqing Medical University authorized all animal experiments that were conducted in accordance with the National Institutes of Health’s guidelines for the use and care of animals (No.2020-790). Here, N-nitro-L-arginine methyl ester, an inhibitor of NO synthase, was used to prepare the stomach to create the PE animal model (L-NAME). Two groups of pregnant mice were randomly assigned (control group, *n* = 6, and L-NAME group, *n* = 6). Mice from the L-NAME group received L-NAME (100 mg/kg body weight) every day between E8.5 and E16.5. The same approach was used to administer saline (1 mL/kg body weight) to the mice in the control group. During E6.5d–E14.5d and E15.5d–E17.5d, tail-cuff plethysmography (BP-2000 Visitech system, Austin, TX, USA) was used to measure blood pressure (BP) every two days. The E17.5d observed the euthanasia of mice. Placentas and fetuses were gathered and weighed.

### 2.3. Cell Culture

The Chinese Academy of Sciences’ Cell Bank provided the JAR and JEG3 cell lines. The HTR8/SVneo cells were purchased from the American Type Culture Collection (ATCC, Manassas, VA, USA). In the Roswell Park Memorial Institute (RPMI)-1640 medium (Gibco, Grand Island, VT, USA) supplemented with 10% fetal bovine serum (FBS, PAN-Biotech, Aidenbach, Germany), 100 U/mL of penicillin, and 100 g/mL of streptomycin (Beyotime, Shanghai, China), both HTR8/SVneo and JAR cells were grown. In order to maintain JEG-3 cells, DMEM/F-12 media (1:1) (Gibco, Grand Island, VT, USA) was supplemented with 10% FBS, 100 U/mL of penicillin, and 100 g/mL of streptomycin. The cells were all kept in an environment of 5% CO_2_ at 37 °C. Recombinant human CCL21 protein (PeproTech, Rocky Hill, CT, USA; 500 ng/mL) was given to trophoblasts to examine its effects.

### 2.4. RNA Extraction and qRT-PCR

A TRIzol reagent was used to extract the total RNA from the human placenta or HTR8/SVneo cells (Invitrogen, Waltham, MA, USA). The purity and concentration of the RNA sample were confirmed by measuring the A260/A280 and A230/260 ratios with NanoDrop (Thermo Fisher Scientific, Waltham, MA, USA). Following this, 1 μg of total RNA from all samples was reverse transcribed using an EvoScript Universal cDNA Master Reagent Kit (Roche, Basel, Switzerland). The qRT-PCR was then carried out with an Applied Biosystems PCR cycler and SYBR green dye (MedChemExpress, Newark, NJ, USA) (Bio-Rad, Hercules, CA, USA). Table S2 showed the primer sequences used in our study (TSINGKE, Beijing, China). As an internal control, β-actin was used. The 2^−∆∆^Ct method was then used to perform the calculation.

### 2.5. Western Blotting

The detailed Western blotting procedure was carried out in accordance with our previous study [20]. The following primary antibodies were used in this study: CCL21 (1:500; Biorbyt, Cambridge, UK); CCR7 (1:1000; ABclonal Technology, Wuhan, China); GAPDH (1:1000; Servicebio, Wuhan, China); E-cadherin (1:1000; ProteinTech, Wuhan, China; Wanleibio, Shenyang, China); N-cadherin (1:1000; Cell Signaling Technology, Danvers, MA, USA); Vimentin (1:1000; ProteinTech, Wuhan, China; Wanleibio, Shenyang, China); Snail (1:1000; Wanleibio, Shenyang, China); β-Actin (1:1000; Cell Signaling Technology, Danvers, MA, USA); Phospho-p44/42MAPK(Erk1/2)^(Thr202/Tyr204)^ (1:2000; Cell Signaling Technology, Danvers, MA, USA); p44/42MAPK(Erk1/2)^(Thr202/Tyr204)^ (1:2000; Cell Signaling Technology, Danvers, MA, USA).

### 2.6. Immunohistochemistry Staining

Before embedding in paraffin, human placental tissues were treated in a 4% paraformaldehyde solution. The slices were then cut into 3–5 μm thick layers. The tissue slices were subsequently dewaxed in xylene and dehydrated in gradient alcohol solutions (100%, 95%, 85%, and 75%) in that order. The slices were then cooked in a microwave for 15 min at medium temperature after being submerged in a sodium citrate buffer for 4 min at high temperature. Following a 10 min soak in 3% H_2_O_2_ drops to neutralize endogenous peroxidase activities, tissue slices were blocked for an hour with caprine serum and then incubated with a CCL21 antibody (1:50; Biorbyt, Cambridge, UK) at 4 °C overnight. Slices were hatched using a secondary antibody that was biotin-labeled (ZSGB-BIO, Beijing, China). The next day, labeling of the target protein and cell nuclei with DAB and hematoxylin in that order. Finally, the stained sections were then observed under a microscope.

### 2.7. Immunofluorescence Staining

The placental tissues were embedded in paraffin after being treated with 4% paraformaldehyde. The tissues were then cut into slices of 3–5 μm thickness. The tissue slides were serially drenched in ethanol and dewaxed in xylene. For antigen retrieval, the slides were then submerged in sodium citrate buffer and warmed in a microwave for 15 min at 50 °C and then 4 min at 100 °C. Following the use of an autofluorescence quenching agent (Sevicebio, Wuhan, China), goat serum was used to block the sample for an additional hour at room temperature. The appropriate antibodies were used to hatch the slides over night at 4 °C. The specific antibodies used in this study were CCL19 (1:50; ABclonal Technology, Wuhan, China); CCL21 (1:100; Aifang Biological, Changsha, China); CCR7 (1:50; ABclonal Technology, Wuhan, China); CK7 (1:50; Aifang Biological, Changsha, China); and HLA-G (1:50; ProteinTech, Wuhan, China). During the following day, the slides were incubated with fluorescein-conjugated secondary antibodies (1:100; Abbkine Biotechnology, Wuhan, China). 4′,6-Diamidino-2-phenylindole was used to stain the nuclei (DAPI, Servicebio Biotechnology, Wuhan, China).

### 2.8. Scratch Wound-Healing Assay

The scratch wound-healing experiment was used to determine the migration ability of HTR8/SVneo cells in the presence or absence of different concentrations of CCL21 (0 ng/mL, 100 ng/mL, 200 ng/mL, and 500 ng/mL). The HTR8/SVneo cells were seeded into 6-well plates and cultured for 24 h. Cell layers were scratched with a 200 μL pipette tip to form a wound-like gap as convergence approached 95%. Cells were then cultured in serum-free RPMI 1640 medium (with or without CCL21) and monitored at 0 h, 24 h, and 48 h intervals. The width of the wound was measured using the Image J program (Rasband, NIH).

### 2.9. Matrigel Invasion and Transwell Migration Assay

The transwell assay in this study was carried out exactly as described in our previous publications. The HTR8/SVneo cell invasion was detected in Matrigel (Corning, New York, NY, USA)-coated transwell inserts (8.0 μm, Corning, New York, NY, USA) with polycarbonate filters full of mini-pores at 8.0 μm. The inserts were pre-coated with Matrigel (diluted with RPMI 1640 incomplete medium at 1:8). After 1h in an incubator at 37 °C relative to the gel, 8 × 10^4^ HTR8/SVneo cells with or without CCL21 were added to the upper chamber, while a 600 μL complete RPMI 1640 medium with or without CCL21 was pipetted into the lower well. The non-invaded cells that remained at the top chambers were swabbed off with a cotton swab after 48 h of incubation for invasion and 24 h for migration. The filters were washed three times with PBS before being fixed in 4% paraformaldehyde for 30 min and dried for 10 min. The inserts were then stained for 20 min with crystal violet. Using the EVOS FL Auto microscope, the trophoblasts in the inserts were examined (Life Technologies, Carlsbad, WA, USA). The cell migration assay was similar to the invasion assay, with the exception that the insert was not coated with Matrigel and the cell count was 4 × 10^4^.

### 2.10. DNA Synthesis Assay

The EdU Kit (RiboBio, Guangzhou, China) was used to perform the 5-ethynyl-2′-deoxyuridine (EdU) assay according to the manufacturer’s instructions. Cells were planted in 96-well plates and then treated after adhesion. Each well received a total of 100 μL of culture medium containing 50 mM EdU. Cells were fixed with 4% formaldehyde for 30 min. After washing, the cells were incubated for 30 min with a solution from the kit before being stained with Hoechst to identify nuclei. A fluorescence microscope was used to capture the images (Thermo Fisher Scientific, Waltham, MA, USA). Image J was used to identify EdU-positive cells.

### 2.11. TUNEL Assay

Cells were seeded in 96-well plates. After 24 h, the cells were incubated with CCL21 for 48 h. The cells were then washed with PBS and fixed for 30 min in 4% paraformaldehyde. A TUNEL assay kit was used to detect apoptotic cells (Beyotime, Beijing, China). Cells were cultured in 0.3% Triton X-100 in PBS for 5 min at room temperature, and then they were cultured in a biotin-labeled solution for 1 h at 37 °C in the dark. Here, DAPI was used to stain the nuclei. An Evos Fl Color Imaging System was used to display the images (Life Technologies, Carlsbad, WA, USA). Each group had three fields chosen at random for analyses.

### 2.12. Cell Immunofluorescence

A total of 2 × 10^4^ cells were seeded into a 24-well plate that had been precoated with cell-crawling tablets. After 48 h of CCL21 treatment, cells were fixed with 4% paraformaldehyde. The cells were then immersed in PBS for 1 h at room temperature before being blocked with BSA. The cells were then incubated overnight at 4 °C with the primary antibody and the corresponding secondary fluorescent antibodies (ProteinTech, Wuhan, China). Here, DAPI was used to stain the nuclei. The images were captured using a fluorescent microscope (Life Technologies, Carlsbad, WA, USA).

### 2.13. Transfection

The sequences used were as follows:

CCR7-siRNA-1#, 5′-GGCUCAAGACCAUGACCGATT-3′;

CCR7-siRNA-2#, 5′-GCGUCCUUCUCAUCAGCAATT-3′;

Negative control siRNA, UUCUCCGAACGUGUCACGUTT.

Here, TSINGKE designed and synthesized the primer sequences (TSINGKE, Beijing, China). Cells were seeded at a density of 2 × 10^5^ cells per well in a 6-well plate, and were transfected with the control siRNA or CCR7 siRNA using the Lipofectamine 2000 reagent (Thermo Fisher Scientific, Waltham, MA, USA) according to the manufacturer’s instructions. Cells were replaced with a fresh complete RPMI 1640 medium after a 6 h transfection. Cells were prepared for qRT-PCR, Western blotting, and other detections 48 h later. The qRT-PCR and Western blotting were used to confirm the transfection efficiency.

### 2.14. Bioinformatic Analysis

#### 2.14.1. Data Acquisition

The GSE75010 dataset was downloaded from the Gene Expression Omnibus (GEO) database (https://www.ncbi.nlm.nih.gov/geo/ (accessed on 13 October 2018)) via R studio (version 4.1.1) and the package “GEOquery”. The expression matrix was annotated by the attached probe annotation matrix. This dataset included the expression profile of 80 PE placentas and 77 non-PE placentas. The differentially expressed genes (DEGs) and gene ontology (GO) enrichment analysis were conducted as previously described [21]. The top 300 differential genes were extracted for gene ontology enrichment analysis by the R package “cluster profiler” [22]. The expression of gene CCR7 was selected for comparison between the preeclampsia and the control group which was visualized by ggplot2.

#### 2.14.2. Gene Set Enrichment Analysis

The results obtained from the DEGs analysis were sorted according to the fold-change, and the ordered matrix was input into a “cluster profiler” for the GSEA enrichment analysis of terms in the gene ontology database [23]. The result was visualized using the R package “ggplot2”.

#### 2.14.3. Blood Pressure Correlation Analysis

Pearson’s correlation analysis was conducted between the CCR7 gene expression value and the maximum systolic and diastolic blood pressure, showing the correlation coefficient R and the corresponding *p*-value. A scatter plot was used to sign each sample, and regression line fitting with respect to the scatter points was performed. Correlation analysis and plotting were performed by using the R package “ggpubr”.

### 2.15. Statistical Analysis

All data were presented as means ± SEM. For comparisons between the two groups, a two-tailed Student’s t-test was used. For comparisons among multiple groups, a one-way ANVOA analysis of variance was used, followed by Tukey’s test. A two-way ANVOA analysis of variance was performed for multiple groups with multiple characteristics. GraphPad Prism 8.0 was used for all statistical analyses (GraphPad Software Inc., La Jolla, CA, USA). If the *p*-value was less than 0.05, the results were considered statistically significant.

## 3. Results

### 3.1. CCR7 Has Been Linked to the Pathogenesis of PE According to Bioinformatics Analysis

**Figure 1:**
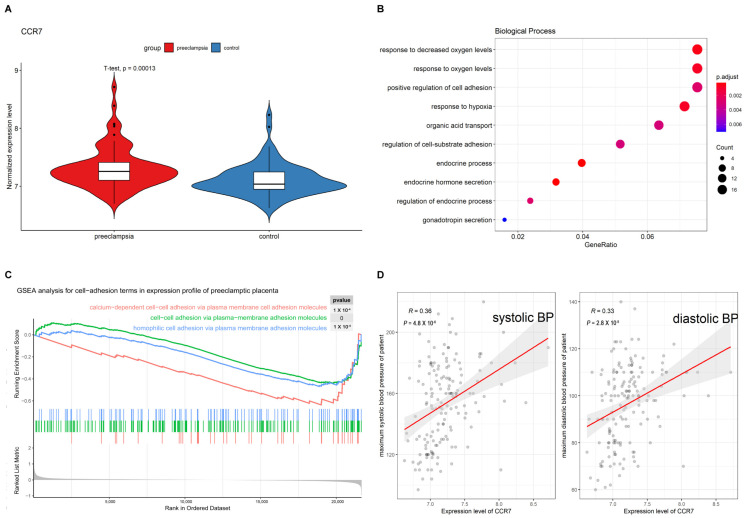
CCR7 is an important gene for normal pregnancy by high throughput analysis. (**A**) Expression violin pattern of CCR7 after differential expression analysis. (**B**) The most significant enriched terms in placentas. (**C**) The hallmark pathways associated with CCR7 in PE placentas. (**D**) Pearson’s correlation coefficient was used to verify the correlation between CCR7 expression level and blood pressure (*p* < 0.05, R1 = 0.36, R2 = 0.33).

First, we downloaded a dataset of preeclamptic placenta tissue from the GEO database. Using the DEGs in the dataset, CCR7 was identified. As presented in Figure 1A, the mRNA expression level of CCR7 was upregulated in the PE group. To further determine the possible mechanism by which CCR7 functioned, the CCR7-related genes were analyzed by GO enrichment analyses. The results demonstrated that the DEGs were mainly involved in mediating cell adhesion and secretion (Figure 1B). The result of the GSEA analysis also showed that cell-cell adhesion was involved in PE, which was in accordance with the GO analysis (Figure 1C). Based on the clinical data in the dataset, Pearson’s correlation coefficient analysis was performed between the CCR7 gene expression value and the maximum systolic and diastolic blood pressure. It revealed that CCR7 was correlated with blood pressure (Figure 1D). It has been indicated that the downregulation of CCR7 suppressed trophoblast migration and invasion [15]. We hypothesized that CCR7 was involved in the pathogenesis of PE based on the results of the bioinformatic analysis.

### 3.2. CCR7 Is Upregulated in the Preeclamptic Placenta

**Figure 2:**
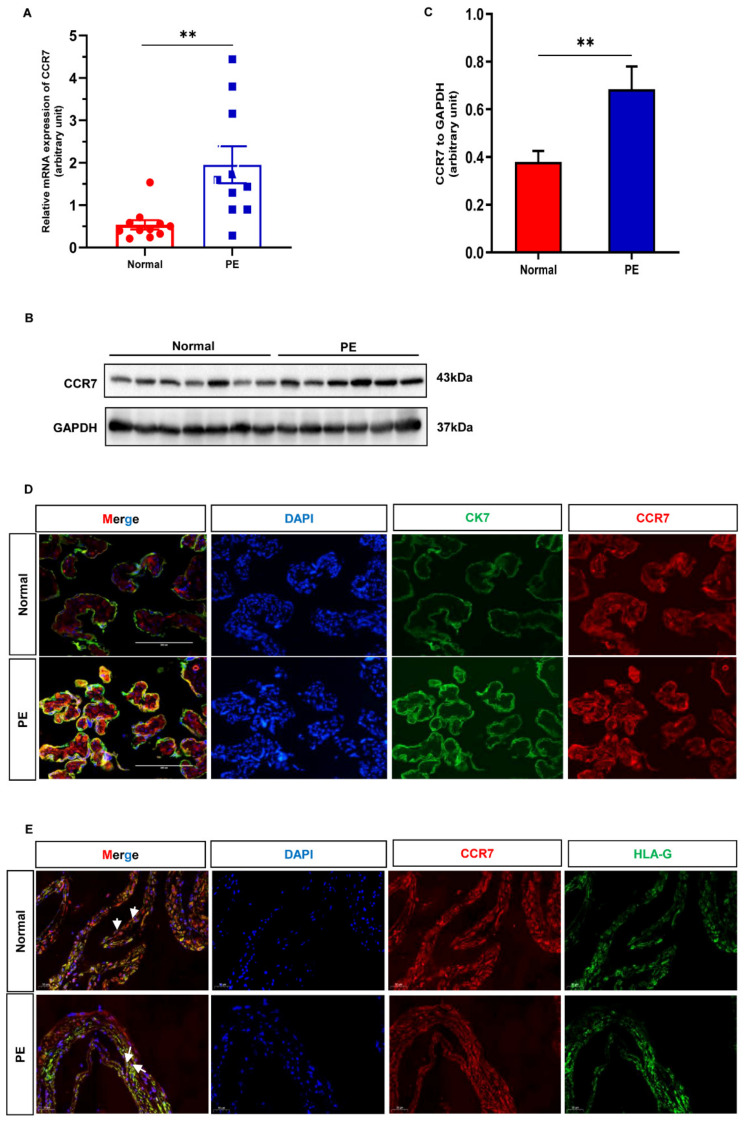
CCR7 expression and localization in human placental tissues and the decidual. (**A**) The transcriptional expression level of CCR7 was analyzed by qRT-PCR in human placental tissues from normal pregnancies (*n* = 11) and PE patients (*n* = 10). (**B**) Western blotting analysis of CCR7 protein expression level in normal (*n* = 7) and PE (*n* = 7) placenta tissues (Supplementary File S1). (**C**) Statistical analysis of protein densitometry quantification in Figure 2B. (**D**,**E**) Double-stained immunofluorescence analysis of CCR7 distribution in the placental and decidual tissues from normal pregnancies and PE patients. Scale bars-200 μm and 50 μm, respectively. The CK7-labeled trophoblast cells and HLA-G-labeled EVT cells are indicated by green signals. The CCR7 protein is indicated by red signals; ** *p* < 0.01.

We gathered placenta tissues from females with normal pregnancies and PE to examine whether CCR7 expression levels were different in the placenta of pregnancies with PE. The qRT-PCR and Western blotting techniques were conducted, respectively. The CCR7 mRNA expression levels were higher in PE placentas than that in normal placentas (Figure 2A), which was in accordance with the results of the bioinformatic analysis. The Western blotting analysis also demonstrated that CCR7 protein expression levels were increased in PE placentas (Figure 2B,C, Supplementary File S1). To further determine the distribution and expression of CCR7, an immunofluorescence analysis was performed in the placenta and decidual. The results showed that CCR7 was co-stained with cytokeratin 7 (CK7) in trophoblast layers in the placenta (Figure 2D). Additionally, CCR7 was expressed in some EVT cells in the maternal decidual, which were identified by the positive expression of human leukocyte antigen G (HLA-G) (Figure 2E). Thus, CCR7 was involved in PE, and we further speculated on the pathophysiological relevance of CCR7 in placental EVT functions.

### 3.3. Expression of CCL21 Is Downregulated in the Placenta of PE

**Figure 3:**
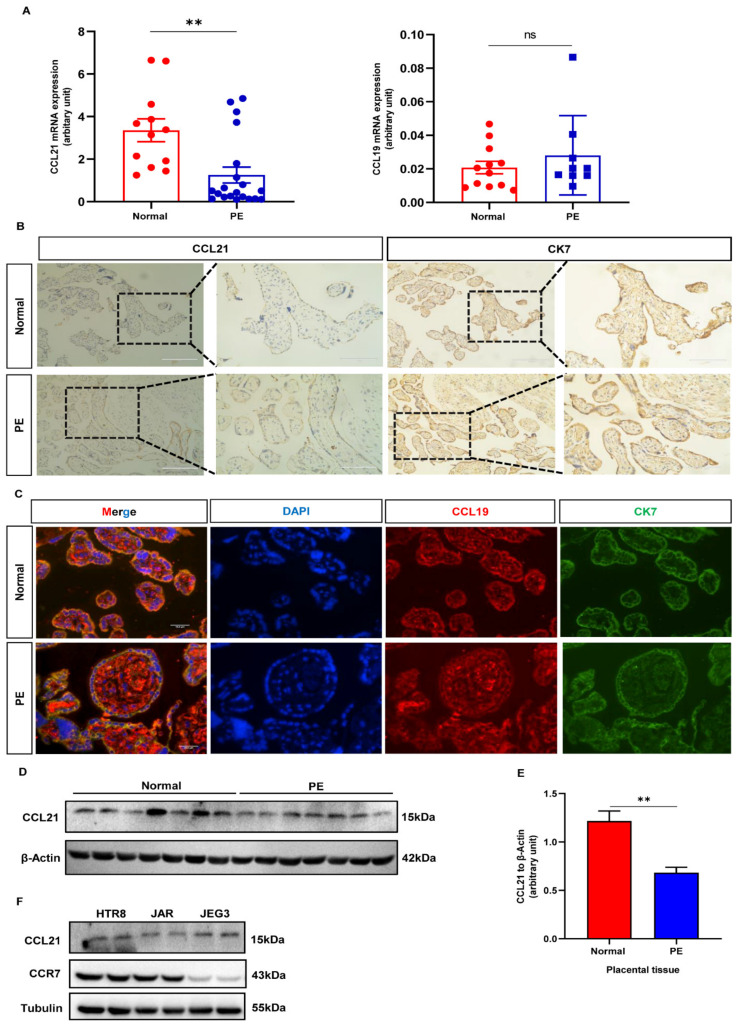
CCL21 and CCL19 expression in human placental tissues from the PE group and the control group. (**A**) Expression of CCL21 and CCL19 mRNA in the PE group (*n* = 20; *n* = 9) and the control group (*n* = 12) by qRT-PCR. (**B**) Immunohistochemistry staining of CCL21 in human placental tissues in the PE group and normal group. Scale bar-100 μm. (**C**) Double-stained immunofluorescence of CCL19 in the human placental tissues in the PE group and normal group. Here, CK7 is indicated by green signals, while CCL19 is indicated by red signals. Scale bar-10 μm. (**D**) Western blotting analysis of CCL21 in normal (*n* = 7) and PE (*n* = 7) placental tissues (Supplementary File S1). (**E**) Statistical analysis of protein densitometry quantification in Figure 3D. (**F**) Western blotting analysis of CCL21 and CCR7 in HTR-8/SVneo, JAR, and JEG3 cells (Supplementary File S1); ** *p* < 0.01; ns, non-significance.

CCL19 and CCL21 have both been identified as CCR7 ligands [24]. However, the CCL19 and CCL21 have yet to be studied at the maternal fetal interface. As a result, the expression patterns of CCL19 and CCL21 were studied. Firstly, qRT-PCR and Western blotting were used to determine the expression levels of CCL19 and CCL21. The results showed that lower levels of CCL21 were found in preeclamptic placentas compared to normal placentas (Figure 3A,D,E, Supplementary File S1). Whereas CCL19 expression levels showed no statistical significance between the normal and PE groups (Figure 3A). Furthermore, placental tissue slides from the third trimester were stained to identify the localization of CCL21 at the maternal fetal interface. The CCL21 protein stained weakly in PE placental syncytiotrophoblasts but moderately in normal placental syncytiotrophoblasts (Figure 3B). The immunofluorescence intensity of the CCL19 protein was the same in both groups (Figure 3C). Therefore, CCL21 was chosen for further investigation. Next, Western blotting was used to detect the expression of CCL21 and CCR7 in three different trophoblast cell lines. The CCR7 was found to be highly expressed in HTR8/SVneo cells and lowly expressed in JEG3 cells. While CCL21 proteins were found at the same level in three trophoblast cell lines (Figure 3F, Supplementary File S1). Collectively, the CCL21 protein is secreted by trophoblast cells, and the CCR7 expression profile suggested that CCL21 might bind to trophoblasts to perform biological functions at the maternal fetal interface.

### 3.4. CCL21 Promotes Trophoblasts Mobility

**Figure 4:**
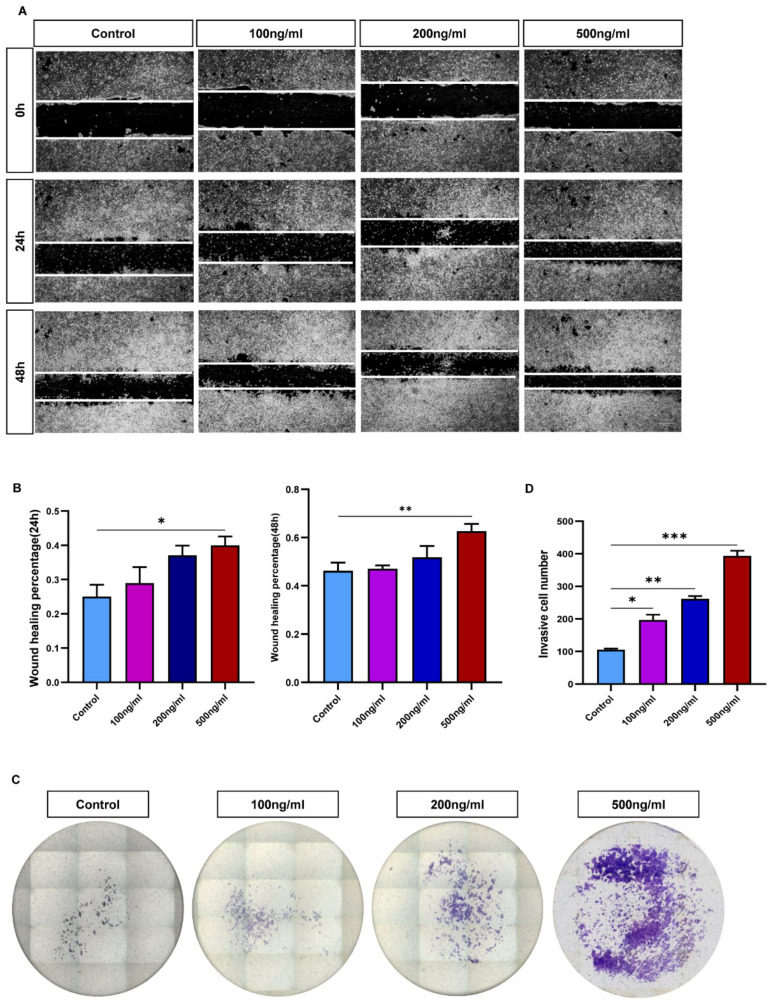
CCL21 promoted trophoblast cell migration and invasion. The HTR8/SVneo cells were treated with 0, 100, 200, and 500 ng/mL of rhCCL21 for 48 h and 24 h, respectively. (**A**,**B**) Scratch wound-healing assay and quantitation were used to determine trophoblast cell migration ability. (**C**,**D**) Matrigel invasion assay and quantitation were performed to investigate trophoblast invasiveness ability; * *p* < 0.05, ** *p* < 0.01, *** *p* < 0.001.

Using HTR8/SVneo cells treated with varying quantities of recombinant human CCL21 protein, scratch wound-healing tests and a transwell assay were carried out to investigate the impact of CCL21 on the migration and invasion of trophoblasts. The findings of the study demonstrated that trophoblast cells treated with rhCCL21 exhibited faster dose-dependent migratory abilities than observed for the control group (Figure 4A,B). Similar to the control group, the rhCCL21-stimulated group showed an increased cell invasion potential in a dose-dependent manner (Figure 4C,D). An EdU assay was also carried out to investigate the impact of CCL21 on trophoblast cell proliferation. The findings demonstrated that CCL21 had no discernible impact on the proportion of proliferating HTR8/SVneo cells in the treatment group compared to the control group (Figure S1A,B). Following exposure to rhCCL21, the apoptosis of HTR8/SVneo cells was discovered using the TUNEL assay and Western blotting. Figure S1C showed that whereas DNase I (positive control) significantly increased cell apoptosis, no significant differences were observed between the treatment and control groups. Western blotting was used to find out more about the effect of CCL21 on apoptosis-related proteins, such as BAX, BCL-2, Caspase 9, and cleaved Caspase 3. These findings also showed that neither group differed significantly from the other (Figure S1D,E, Supplementary File S1). These findings suggested that CCL21 could significantly improve the migration and invasion of trophoblasts in vitro.

### 3.5. CCL21-Induced Trophoblasts Migration and Invasion Are Dependent on the EMT Process

**Figure 5:**
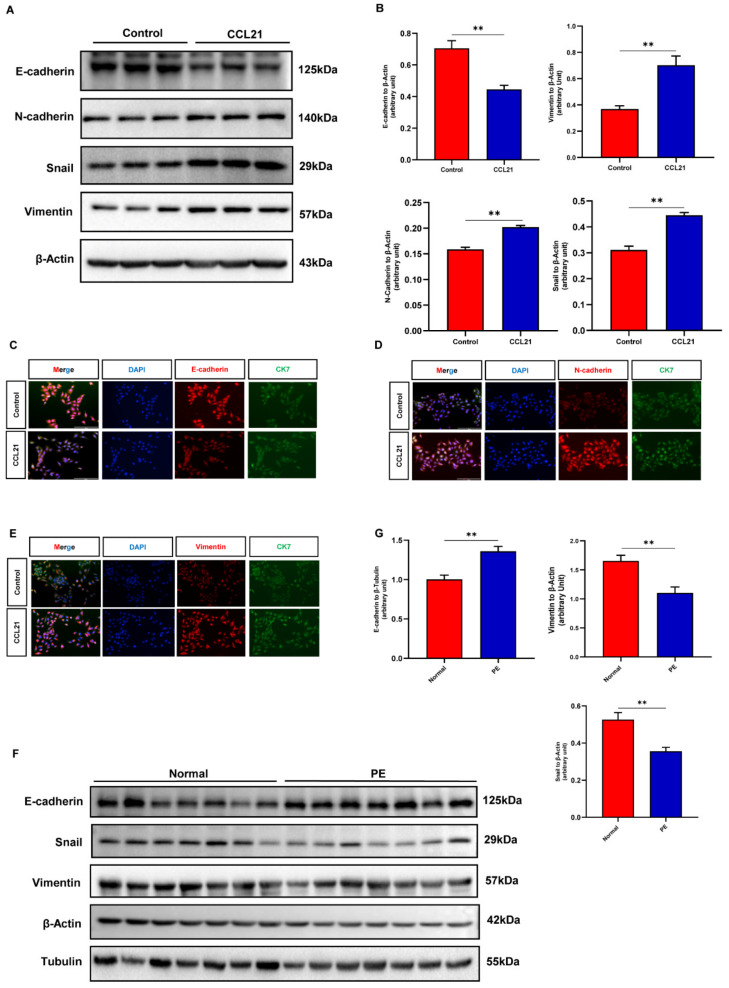
CCL21 contributed to the epithelial-to-mesenchymal transition process of HTR8/SVneo cells. The HTR8/SVneo cells were treated with 500 ng/mL of rhCCL21 for 48 h in a medium containing 0.5% FBS. (**A**) EMT biomarkers were detected by Western blotting between the treatment and the control groups (Supplementary File S1). (**B**) Statistical analysis of protein densitometry quantification in Figure 6A. (**C**–**E**) Cell immunofluorescence was used to measure the expression of EMT markers. Here, CK7 is indicated by green signals; E-cadherin, N-cadherin, and vimentin are indicated by red signals. (**F**) Western blotting was performed to analyze the expression of EMT markers in placental tissues from the PE patients and normal pregnancies (Supplementary File S1). (**G**) Statistical analysis of protein densitometry quantification in Figure 6F; ** *p* < 0.01.

It has been reported that EMT was thought to be a crucial step in how trophoblasts acquired their capacity for invasion [12]. The expression of EMT markers was discovered in order to determine if CCL21 caused trophoblast migration and invasion via the EMT mechanism. According to the results of Western blotting, exposure to CCL21 increased the expression of the mesenchymal markers N-cadherin, vimentin, and Snail in HTR8/SVneo cells while decreasing the expression of the epithelial marker E-cadherin (Figure 5A,B, Supplementary File S1). Additionally, identical phenomena to those observed in the Western blotting results were also observed in the results of cell immunofluorescence (Figure 5C–E). By using Western blotting, we were also able to identify the expression of E-cadherin, vimentin, and Snail in placental tissues. The findings showed that, when compared to the control groups, placental tissues from PE patients expressed more E-cadherin while expressing less vimentin and Snail (Figure 5F,G, Supplementary File S1).

### 3.6. CCL21 Affects the EMT Process in a Receptor-Dependent Manner and Further Affects the Migration and Invasion of Trophoblasts

**Figure 6:**
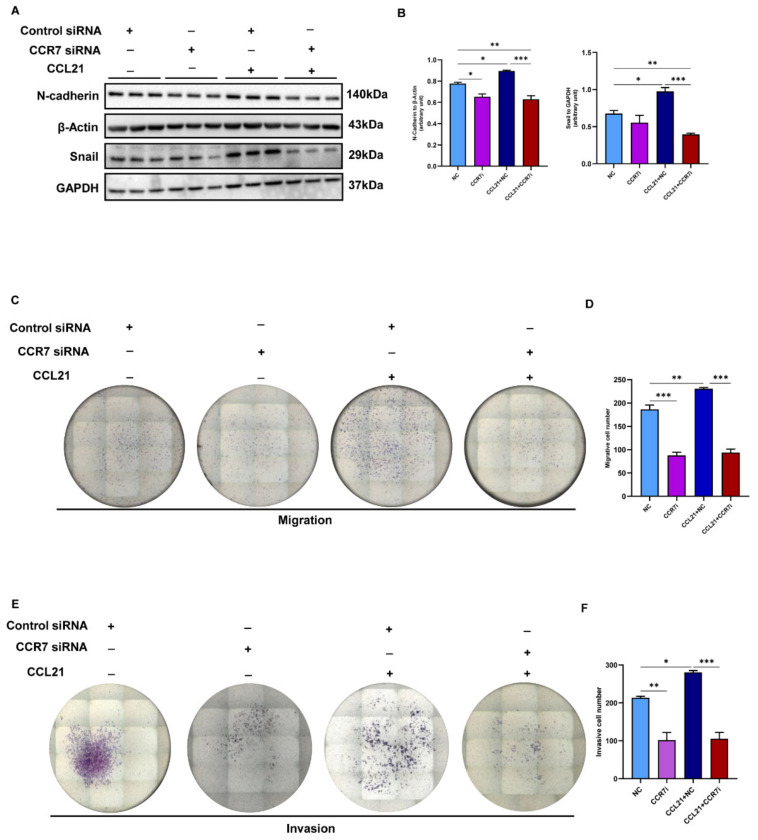
Knockdown of CCR7 inhibited trophoblast cells EMT, migration, and invasion. The HTR8/SVneo cells were transfected with small interfering CCR7. After 48 h transfection, the cells were treated with 500 ng/mL of rhCCL21 for 48 h in a medium containing 0.5% FBS. (**A**) Western blotting analysis of the expression of EMT markers in CCR7-knocked-down cells treated with rhCCL21 or not (Supplementary File S1). (**B**) Statistical analysis of protein densitometry quantification in Figure 7A. (**C**,**D**) Scratch wound-healing assay and quantitation were performed to determine trophoblast cell migration ability. (**E**,**F**) Matrigel invasion assay and quantitation were conducted to investigate trophoblast invasiveness ability; * *p* < 0.05, ** *p* < 0.01, *** *p* < 0.001.

We then determined whether CCL21 had an impact on the EMT, invasion, and the migration of trophoblasts via its receptor, since CCR7 was expressed in placental tissues. The small interfering CCR7 was transfected into HTR8/SVneo cells to investigate the function of CCR7 in CCL21-enhanced trophoblast EMT, migration, and invasion. The CCR7 was markedly downregulated at the mRNA and protein levels, according to qRT-PCR and Western blotting, respectively (Figure S2A–C, Supplementary File S1). The effects of CCL21 on trophoblast cell EMT, migration, and invasion were then examined in CCR7-knocked-down cells. The Western blotting results showed that the expression levels of EMT markers were lower than those in the NC cells after the CCR7-knocked-down cells were stimulated by CCL21 (Figure 6A,B, Supplementary File S1). Additionally, CCR7 knockdown inhibited CCL21-stimulated HTR8/SVneo cell migration and invasion (Figure 6C–F). These findings showed that CCL21′s binding to CCR7 allows it to control trophoblast EMT, migration, and invasion. The CCR7 knockdown significantly affected the trophoblast EMT, migratory, and invasion alterations brought on by CCL21.

### 3.7. CCL21/CCR7 Axis Activates the ERK1/2 Signaling Pathway to Induce EMT in Trophoblasts

**Figure 7:**
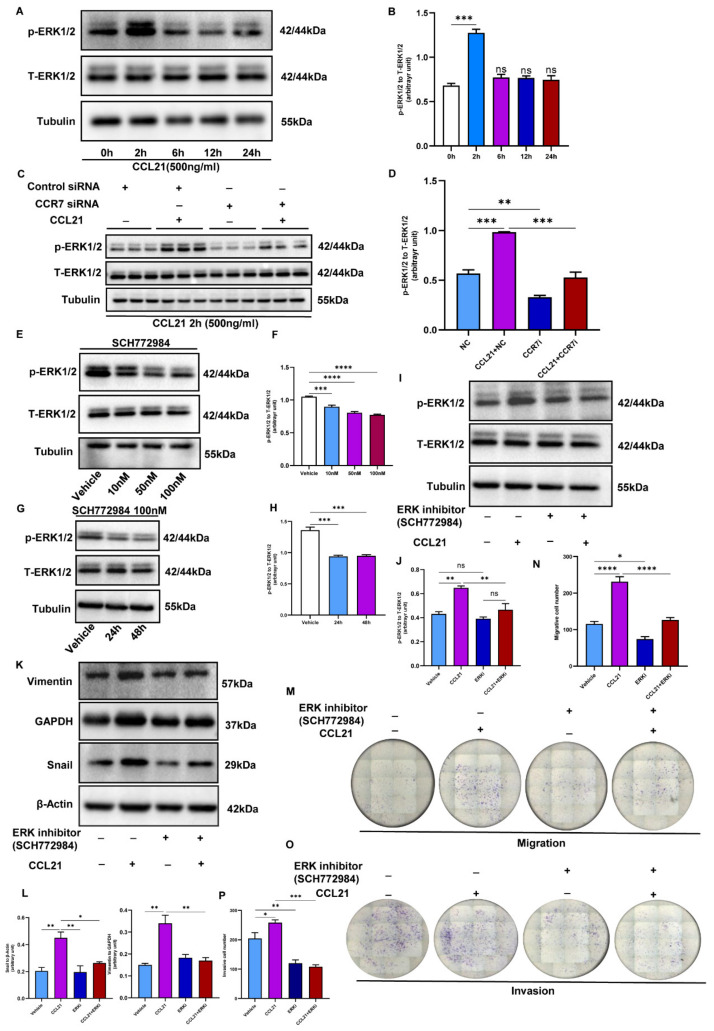
CCL21/CCR7 activates the ERK1/2 signaling pathway to promote the EMT process, migration, and invasion of trophoblast cells. The HTR8/SVneo cells were pretreated with SCH772984 (an ERK1/2 inhibitor) before exposure to rhCCL21. (**A**,**B**) Western blotting analysis revealed the activation of the ERK1/2 pathway in HTR8/SVneo cells stimulated by rhCCL21 for 2 h, 6 h, 12 h, and 24 h (Supplementary File S1). (**C**,**D**) Western blotting analysis showed a blocking effect of the ERK1/2 pathway in CCR7-knockdown HTR8/SVneo cells (Supplementary File S1). (**E**,**F**) ERK1/2 phosphorylation in HTR8/SVneo cells in response to various doses (10–100 nM) of SCH772984 as detected by Western blotting (Supplementary File S1). (**G**,**H**) The phosphorylation of ERK1/2 in HTR8/SVneo cells in response to SCH772984 (100 nM) treatments over a 48h time course. (**I**,**J**) The effects of SCH772984 (100 nM) treatment on phosphorylation ERK1/2 were determined by Western blotting (Supplementary File S1). (**K**,**L**) Western blotting was used to examine the expression of vimentin and Snail in HTR8/SVneo cells treated with SCHCH772984 for 48 h (Supplementary File S1). (**M**,**N**) A transwell migration assay was performed to detect the trophoblast migration ability after exposure to SCH772984 for 24 h. (**O**,**P**) A Matrigel invasion assay was used to examine the trophoblast invasion ability after stimulation with SCH772984 for 24 h; * *p* < 0.05, ** *p* < 0.01, *** *p* < 0.001, **** *p* < 0.0001, ns, non-significance.

The activity of the ERK1/2 signaling pathway was tested to investigate the potential mechanism by which CCL21 induced EMT, migration, and invasion in trophoblasts. Previous research has shown that the CCL21/CCR7 axis promotes EMT and metastasis in cancer cells by activating the MEK/ERK1/2 signaling pathway [16,25]. Following previous research, the Western blotting analysis revealed that the phosphorylation ERK1/2 expression increased in CCL21-treated trophoblasts, while the total ERK1/2 levels remained unchanged (Figure 7A,B, Supplementary File S1). Additionally, the expression of phosphorylation ERK1/2 also remarkably decreased in CCR7 knockdown cells handled by CCL21 (Figure 7C,D, Supplementary File S1). After that, a specific inhibitor of an ERK1/2 signaling pathway (SCH772984) was selected for further investigation. Firstly, various dosages of the ERK1/2-specific inhibitor (SCH772984) were added to HTR8/SVneo cells for different time periods, and dosages greater than 10 nM and exposure time over 24 h significantly inhibited ERK1/2 phosphorylation (Figure 7E–H, Supplementary File S1). Next, the inhibitor pretreatment was conducted 24 h before CCL21 exposure in HTR8/SVneo cells. The results indicated that SCH772984 pretreatment remarkably inhibited the effect of CCL21 on ERK1/2 activation (Figure 7I,J, Supplementary File S1). Furthermore, SCH772984 pretreatment reduced vimentin and Snail expression in HTR8/SVneo cells compared to CCL21 alone (Figure 7K,L, Supplementary File S1), indicating that SCH772984 reversed the CCL21-induced EMT process. Furthermore, the non-Matrigel migration assay and Matrigel invasion assay analyses revealed that the ERK1/2 signaling pathway inhibited migratory and invasive abilities (Figure 7M–P). In summary, these findings confirmed that CCL21 activated the ERK1/2 pathway, causing EMT and promoting trophoblast migration and invasion.

### 3.8. Role of CCL21/CCR7 Axis to Induce EMT in the PE Mouse Model

**Figure 8:**
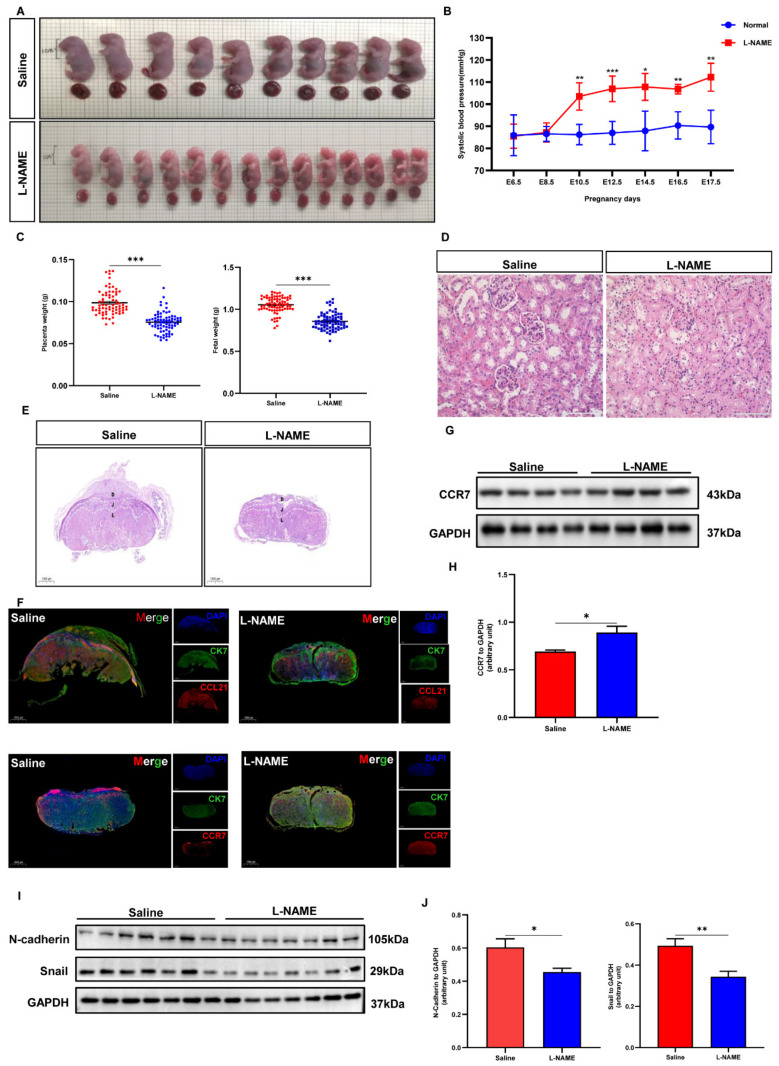
Altered expression of CCL21, CCR7, and EMT biomarkers in mice with L-NAME-induced PE model. (**A**) Representative images of the fetuses of the saline group and the L-NAME group. Scale bar-1 cm. (**B**) Systolic blood pressure of pregnant mice in the saline group and the L-NAME group (*n* = 6). (**C**) The weights of the 75 embryos and 70 placentas from 6 dams in the saline group, and the 73 embryos and 73 placentas from 6 dams in the L-NAME group were measured. (**D**,**E**) Hematoxylin eosin-stained maternal kidney and placenta sections of saline- or L-NAME-treated mice at E17.5d. Scale bar-100 μm; 1000 μm. Abbreviations are as follows: D, decidual; J, junction zone; L, labyrinth. (**F**) Immunofluorescence of CCL21 and CCR7 in the mouse placental tissues in the L-NAME group and the saline group at E17.5d. Here, CK7 is indicated by green signals; CCL21 and CCR7 are indicated by red signals. Scale bar-1000 μm. (**G**–**J**) Western blotting analysis of CCR7 and EMT biomarkers in placenta collected from the saline group and the L-NAME group at E17.5d and quantification (Supplementary File S1); * *p* < 0.05, ** *p* < 0.01, *** *p* < 0.001.

We further investigated the expression of CCL21, CCR7, and EMT biomarkers in mice with an L-NAME-induced PE model (Figure 8A). The mean systolic blood pressure (SBP) of the saline group on E8.5d was 86.96 ± 3.263 mmHg and that of pregnant mice awaiting L-NAME treatments was 87.2 ± 4.346 mmHg. After treatment with L-NAME, the mean SBP was increased when compared with the saline group (Figure 8B). However, the average weights of placental tissues and fetuses in the L-NAME-induced PE group were lower than those in the saline group (Figure 8C). The hematoxylin eosin staining of the kidney showed a decrease in the area of open capillaries in the glomerulus, as well as glomerular atrophy (Figure 8D). The hematoxylin eosin staining of the placental tissues revealed a disordered placental structure and a reduction in the labyrinth area (Figure 8E). The abnormal decrease in placental functional regions indicated that L-NAME led to abnormal placental developments. These findings indicated that L-NAME can cause a PE-like phenotype in mice. The IF and Western blotting analyses were performed to validate the in vitro results in an in vivo manner. Here, CCR7′s protein expression was observed to be upregulated in PE mouse placental tissues, while CCL21 was found to be downregulated (Figure 8F–H). Western blotting was also used to determine the EMT biomarkers (N-cadherin and Snail). The results showed that the expression of N-cadherin and Snail was lower in the PE group compared to the saline group (Figure 8I,J). The findings of the animal study were consistent with those found in human samples and HTR8/SVneo cells. These findings support CCL21/CCR7-mediated EMT regulation in the pathogenic process of PE.

## 4. Discussion

The diagnosis and treatment of PE received substantial attention as a representative of critical obstetrics [26]. The most well-known pathogenesis is incomplete spiral artery remodeling caused by impaired extravillous trophoblast infiltration [27]. Chemokines have been considered to be associated with the angiogenesis and metastatic potential of tumors. A number of chemokines and their receptors have been identified at the maternal fetal interface, implying that they may play an important role in placentation [28]. In this study, we found that CCL21 expression was downregulated in the PE group. An in vitro study indicated that CCL21 could promote the trophoblast EMT process and further enhance its migration and invasion ability. In addition, the biological functions of CCL21 on trophoblasts via the ERK1/2 signaling pathway were confirmed by using a specific inhibitor of this signaling. Finally, with the established PE mouse model, we obtained similar results, with the downregulation of CCL21 and EMT biomarkers, and the upregulation of CCR7. These findings suggest that the abnormal CCL21/CCR7 activation of the ERK1/2 signaling pathway influences EMT and trophoblast migration and invasion.

Chemokines are multifunctional molecules that play roles in cellular communication and signaling. By binding to GPCRs, chemokines can regulate cell-directed migration (chemotaxis), adhesion, cell localization, and cell–cell interactions. The chemokine system has been widely identified in the field of cancer and is involved in tumor invasion, angiogenesis, and metastasis [29]. According to one study, CCL21/CCR7 played an important role in human colon cancer metastasis [30]. Another study discovered that CCL21/CCR7 promoted esophageal squamous cell carcinoma lymph node metastasis [31]. Trophoblasts demonstrated striking similarities to cancer cells [32]. An abundant chemokine network has recently been discovered at the maternal–fetal interface, where it performs its main biological functions in immune tolerance and trophoblast invasion during pregnancy [33]. Previous research found a link between chemokines and trophoblast mobility. The CCL24 promoted the growth and invasiveness of trophoblast cells in vitro via the ERK1/2 and PI3K signaling pathways, according to Li et al. [34]. The CXCL12 induced trophoblast cell invasion by regulating MMP2 and MMP9, according to Zhou et al. [35]. Additionally, CX3CL1, CCL14, and CCL4 have demonstrated outcomes that are comparable [36]. As a result, this study investigated how CCL21/CCR7 affects trophoblast function. First, placental tissues taken from patients with PE and controls were found to express CCL21 and CCR7. We discovered that CCL21 was downregulated, and that its receptor, CCR7, was upregulated in preeclamptic placentae, which was consistent with the findings of carotid atherosclerosis [37]. A mouse model of PE was developed to test the theory in vivo. It is possible to induce PE in pregnant mice by administering L-NAME, a NOS inhibitor. The experimental results, such as hypertension, renal damage, and intrauterine growth limitation, are comparable to those in human PE [38,39]. According to the PE mouse model data, CCL21 was downregulated and CCR7 was upregulated, which is consistent with the clinical samples. The compensatory restoration of the impaired trophoblast activities is most likely the cause of the overexpression of CCR7. Levels of CCR7 were reportedly shown to be increased in the placenta of patients with PE. Our findings were in agreement with a recent study that demonstrated that CCR7 promoted invasion and migration in trophoblasts [15]. It is commonly acknowledged that malplacentation, which causes ischemia and hypoxia, can be caused by defective trophoblast invasion and spiral artery remodeling. According to one study, low oxygen supplies in the placenta can upregulate the expression of CCR7 [40], which could form a compensatory mechanism to ensure normal pregnancy progression. Following that, we confirmed the effects of the CCL21/CCR7 axis in vitro. CCL21/CCR7 coactions were found to promote trophoblast invasion and migration. The downregulation of CCL21/CCR7 results in insufficient trophoblast invasion and the development of PE.

Here, EMT is a physiological process that occurs when epithelial cells lose their polarity and acquire a mesenchymal phenotype [41]. It is classified into three types, as follows: (1) embryonic development and organogenesis; (2) wound healing and organ fibrosis; (3) cancer progression. A growing body of research provided strong evidence that EMT plays an important role in cancer progression and metastasis in many types of malignancies over the last decade. Tumor cells undergo tight junction lysis, apical basal polarity disruption, and cytoskeletal restructuring during EMT, allowing the development of an aggressive phenotype. Extracellular stimuli from the tumor microenvironment, such as growth factors, chemokines, and inflammatory cytokines, as well as physical stress within the tumor, regulate EMT in cancer cells. As a result of EMT programming, tumor cells can adapt to their constantly changing microenvironment and successfully metastasize [42]. Similarly, trophoblasts undergo partial EMT during placental development, losing their organized epithelial phenotype and gaining a mesenchymal phenotype, allowing their migration and infiltration into the maternal decidua and vessels [12,43]. According to Pang et al., the CCL21/CCR7 axis mediated TGF-β1-induced EMT and migration in breast cancer [44]. Zhang et al. demonstrated that the CCL21/CCR7 axis facilitated pancreatic cancer metastasis via the EMT and the ERK/NF-κB pathway [16]. Furthermore, CCL21/CCR7 induced EMT in chondrosarcoma by upregulating slug signaling, according to Li et al. [45]. As a result, we looked into whether the CCL21/CCR7 axis was involved in the EMT process in trophoblasts. We found evidence that trophoblasts underwent EMT at the molecular and morphological levels after being exposed to CCL21. The CCL21-treated cells demonstrated improved invasion and migration abilities. Chemokines must bind to their specific receptor in order to have an effect. The small interfering RNA was then used to knock down CCR7 in trophoblasts. The CCR7 knockdown cells almost had no response to CCL21. Following that, we looked at the results from human samples and HTR8/SVneo cells in the L-NAME-induced PE mouse model. The findings in the PE mouse model were consistent with the findings in the human study and HTR8/SVneo cells. These findings suggested that CCL21 facilitated EMT by binding to CCR7.

Extracellular signal-regulated kinase (ERK) signaling pathway is an important core signaling pathway. The activation of receptors, such as receptor tyrosine kinases (RTKs), initiates ERK signaling. The dual phosphorylation of ERK is activated via a series of cascade reactions. As a result, ERKs stimulate kinase activity and catalyze the phosphorylation of a variety of substrates involved in cell proliferation, differentiation, cell motility, and tumorigenesis [46]. In recent years, it has been discovered that the ERK signaling pathway plays an important role in regulating trophoblast cell migration and invasion. The EIF5A1 induced trophoblast migration and invasion via the ERK signaling pathway, according to Zhang et al. [47]. Cyclosporine A was found to induce actin expression and enhanced the proliferative and invasive potential of trophoblasts via MAPK/ERK signaling, according to Du et al. [48]. Furthermore, a recent study found that let-7b influenced trophoblast EMT via ERK signaling [49]. Another study found that calcitriol aided trophoblast invasion by inducing EMT via the ERK signaling pathway [9]. However, it is unknown whether CCL21/CCR7 can activate the ERK signaling pathway to regulate EMT in trophoblasts. The current findings confirmed that CCL21 significantly increased the expression of p-ERK1/2 in HTR8/SVneo cells. The expression of EMT-related markers was reduced when the ERK signaling pathway was inhibited with a specific inhibitor, namely SCH772984. Furthermore, SCH772984 also inhibited the migration and invasion abilities induced by CCL21/CCR7. These findings suggested that the ERK signaling pathway was involved in CCL21/CCR7-regulated trophoblast EMT.

## 5. Conclusions

In conclusion, the findings of this study show that the CCL21/CCR7 axis activates the ERK1/2 signaling pathway to promote EMT and enhances trophoblast migration and invasion. This provides us with more information about the molecular pathogenesis of PE and contributes to effective therapeutic strategies.

## Data Availability

The datasets generated during the current study are available upon reasonable request from the corresponding author.

## Supplementary Materials

The following supporting information can be downloaded at: https://www.mdpi.com/article/10.3390/biology12020150/s1, Figure S1: CCL21 had no obvious effects on proliferation and apoptosis of HTR8/SVneo cells.; Figure S2: Expression of CCR7 after transfection with small interfering RNA. Table S1: Clinical characteristics of study objects; Table S2: Characteristics of primers. Supplementary File S1: The original Western blot figures. The antibodies used to detect the target protein were as follows: CCL21 (~15 kDa, orb136282; 1:500; Biorbyt, Cambridge, UK); CCR7 (~43 kDa, A0121; 1:1000; ABclonal Technology, Wuhan, China); GAPDH (~37 kDa, GB12002; 1:1000; Servicebio, Wuhan, China); E-cadherin (~125 kDa, 20874-1-AP; 1:1000; ProteinTech, Wuhan, China); N-cadherin (~140 kDa, 13116; 1:1000; Cell Signaling Technology, Danvers, MA, USA); Vimentin (~57 kDa, 10366-1-AP; 1:1000; ProteinTech, Wuhan, China); Snail (~29 kDa, WL01863; 1:1000; Wanleibio, Shenyang, China); β-Actin (~42 kDa, 8457; 1:1000; Cell Signaling Technology, Danvers, MA, USA); Phospho-p44/42MAPK(Erk1/2)(Thr202/Tyr204) (~42/44 kDa, 4370T; 1:2000; Cell Signaling Technology, Danvers, MA, USA); p44/42MAPK(Erk1/2)(Thr202/Tyr204) (~42/44 kDa, 4695T; 1:2000; Cell Signaling Technology, Danvers, MA, USA); Tubulin (~55 kDa, GB122667; 1:1000; Servicebio, Wuhan, China); β-Actin (~42 kDa, 8457; 1:1000; Cell Signaling Technology, Danvers, MA, USA); BAX (~20 kDa, 2772; 1:1000; Cell Signaling Technology, Danvers, MA, USA); BCL-2 (~26 kDa, 3498; 1:1000; Cell Signaling Technology, Danvers, MA, USA); Caspase 9 (~20 kDa, WL03421; 1:1000; Wanleibio, Shenyang, China); cleaved Caspase 3 (~20 kDa, WL02117; 1:1000; Wanleibio, Shenyang, China).
