# Supplementary material for: CCL21/CCR7 Axis Contributes to Trophoblastic Cell Migration and Invasion in Preeclampsia by Affecting the Epithelial Mesenchymal Transition via the ERK1/2 Signaling Pathway
Source: Biology (Basel). 2023 Jan 18;12(2):150. doi: 10.3390/biology12020150 (PMC9952405; doi:10.3390/biology12020150)
Supplement: Supplementary file 1 [file biology-12-00150-s001.zip › Supplementary/Supplementary figures and tables.docx]

**CCL21/CCR7 Axis Contributes to Trophoblastic Cell Migration and Invasion in Preeclampsia by Affecting the Epithelial Mesenchymal Transition Via the ERK1/2 Signaling Pathway**

Zheng Liu^1,2^, Jie He^1,2^, Pingsong Jin^1,2^, Yuxin Ran^3^, Nanlin Yin^2,4*^ and Hongbo Qi^1,2,3*^

1Department of Obstetrics, The First Affiliated Hospital of Chongqing Medical University, Chongqing 400016, China

2 Chongqing Key Laboratory of Maternal and Fetal Medicine, Chongqing Medical University, Chongqing 400016, China

3 Department of Obstetrics, Women and Children’s Hospital of Chongqing Medical University, Chongqing 401147, China

4 Department of Center for Reproductive Medicine, The First Affiliated Hospital of Chongqing Medical University, Chongqing 400016, China

*Corresponding author: Nanlin Yin (E-mail: yinnanlin@cqmu.edu.cn); Hongbo Qi (E-mail: qihongbocy@gmail.com)

| Table S1. Clinical characteristics of study objects | | |
| --- | --- | --- |
| Maternal characteristics | Normal pregnancy  (n=20) | Preeclampsia  (n=20) |
| Maternal age (years) | 29.00±2.66 | 29.75±3.71^a^ |
| Gestational age (weeks) | 39.35±0.77 | 36.59±1.25^b^ |
| Pre-pregnancy BMI (kg/m^2^) | 20.41±2.49 | 20.62±1.57^a^ |
| Systolic blood pressure (mm Hg) | 114.10±11.55 | 160.50±10.46^b^ |
| Diastolic blood pressure (mm Hg) | 73.10±9.45 | 100.80±7.34^b^ |
| Neonatal birth weight (g) | 3361±255.60 | 2480±501.60^b^ |

*Abbreviation: BMI, body mass index.*

*Data are presented as mean ± SD. a ns, b P < 0.0001.*

| Table S2. Characteristics of primers |
| --- |
| Genes Sense primer (5’ → 3’) Anti-sense primer (5’ → 3’) |
| CCL19 GGAGTCCGAGTCAAGCATTGT AACACCAGGCGGCTTTATTG |
| CCL21 AAGGCAGTGATGGAGGGGCT GCGGGGCAAGAACAGGATAG  CCR7 GTGTGGGCATCTGGATACTAGC GGTGCGGATGATGACAAGGTA  β-Actin TGGCACCCAGCACAATGAA CTAAGTCATAGTCCGCCTAGAAGCA |

**Figure S1.**


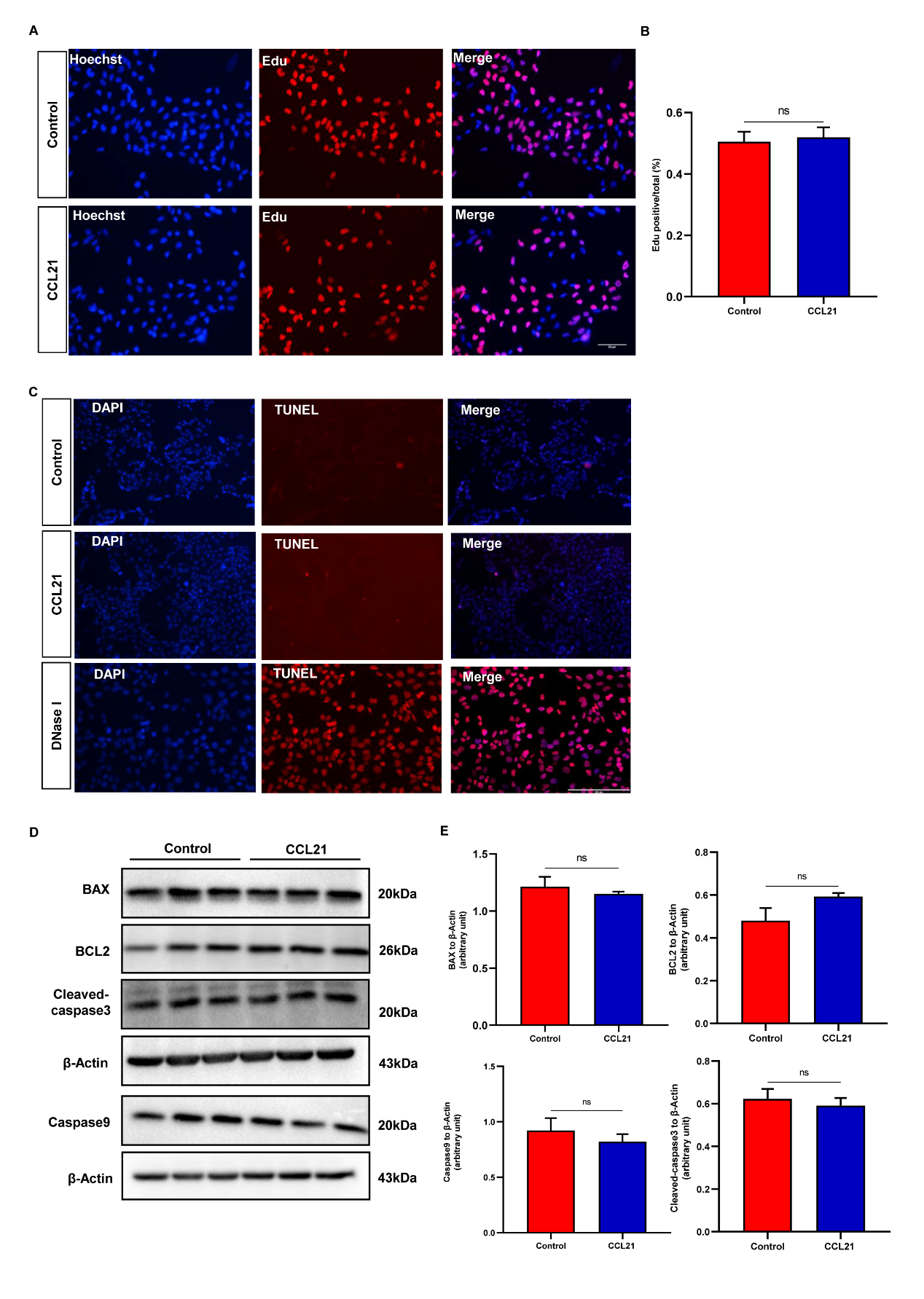


Figure S1. CCL21 had no obvious effects on proliferation and apoptosis of HTR8/SVneo cells. HTR8/SVneo cells were serum-starved for 12h and then treated with 500 ng/ml of rhCCL21 for 48h. (A, B) Cell proliferation was measured with 5-ethynyl-20-deoxyuridine incorporation assay. The blue color indicates the nuclei, and the red color represents the Edu-positive nuclei. Scale Bar: 200 μm. (C) The TUNEL assay kit was used to analyze the number of apoptotic cells between the CCL21-treated group and the control group. The blue color indicates the nuclei, and the red color represents the apoptotic cell. DNase I is used as the positive control. Scale bar: 200 μm. (D) The apoptosis-related protein biomarkers were measured by western blotting between the treatment and the control groups. (E) Statistical analysis of protein densitometry quantification in Figure S1D. ns, non-significance.

**Figure S2.**


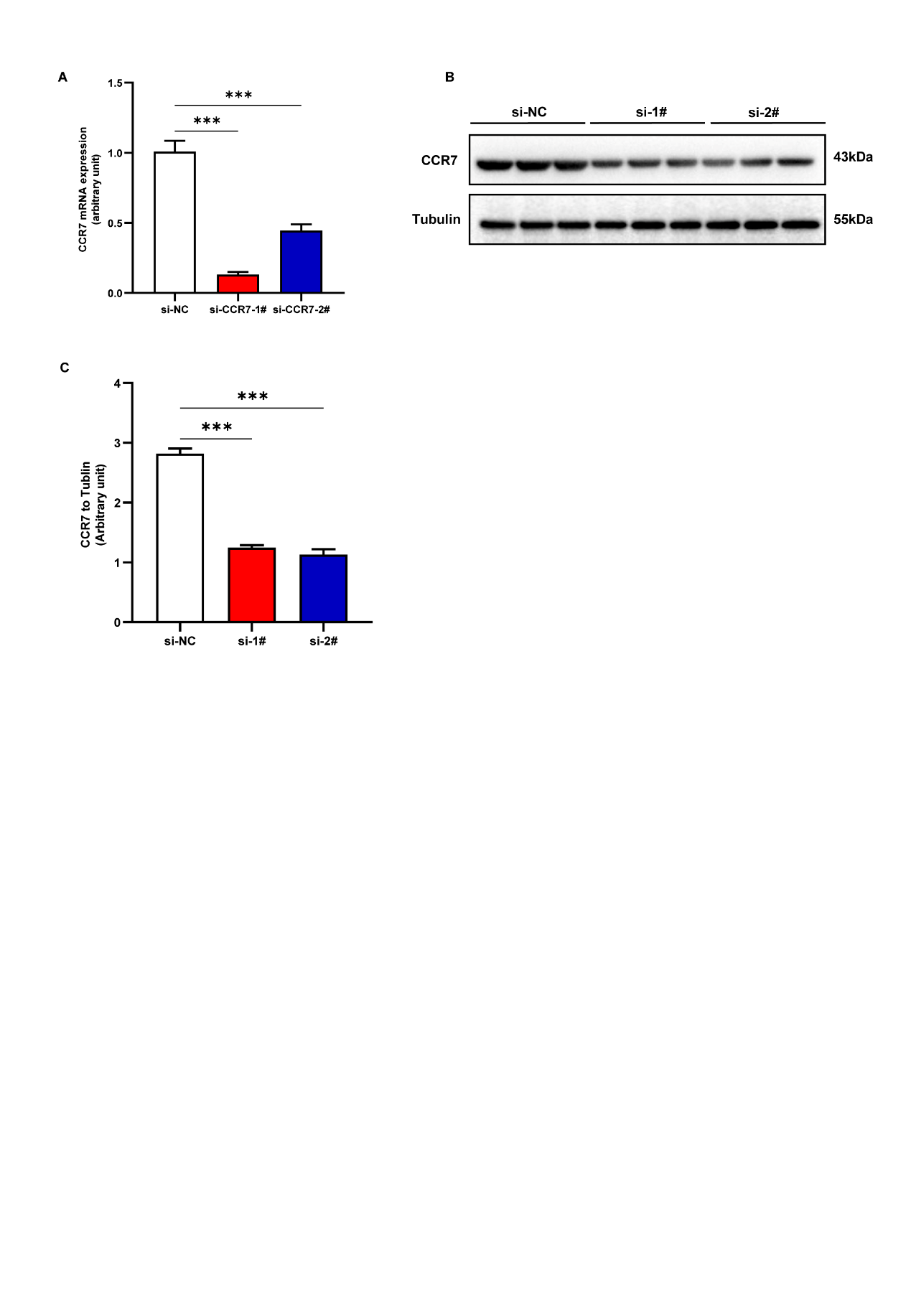
Figure S2. Expression of CCR7 after transfection with small interfering RNA. (A) mRNA expression level of CCR7 was detected by qRT-PCR after transfection with small interfering RNA for 48h. (B, C) Western blotting analysis of CCR7 after transfection with small interfering RNA for 48h and quantification. ***p<0.001.
