# Supplementary material for: CCL21/CCR7 Axis Contributes to Trophoblastic Cell Migration and Invasion in Preeclampsia by Affecting the Epithelial Mesenchymal Transition via the ERK1/2 Signaling Pathway
Source: Biology (Basel). 2023 Jan 18;12(2):150. doi: 10.3390/biology12020150 (PMC9952405; doi:10.3390/biology12020150)
Supplement: Supplementary file 1 [file biology-12-00150-s001.zip › Supplementary/Supplementary File S1.pdf]

Figure 2B

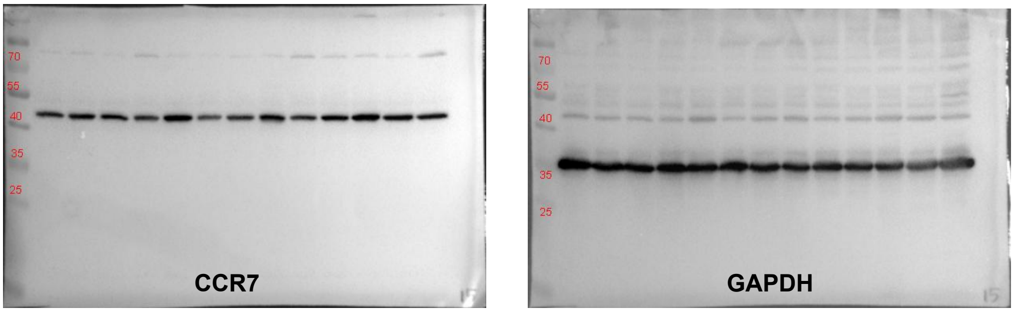

Control	PE
0.291248	0.641928
0.456164	0.391904
0.397083	0.602876
0.26815	1.011612
0.597102	0.731406
0.259087	0.435777
0.388753	

Figure 3D

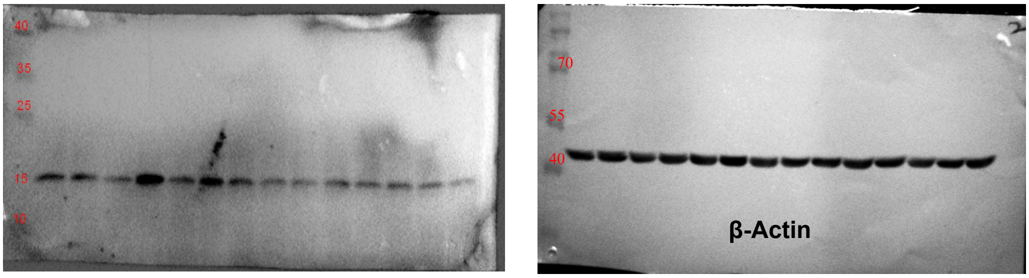

Normal	PE
1.28565	0.56815
1.03117	0.552082
0.921357	0.797873
1.703261	0.68447
1.028508	0.898443
1.410713	0.777093
1.135657	0.495765

Figure 3E

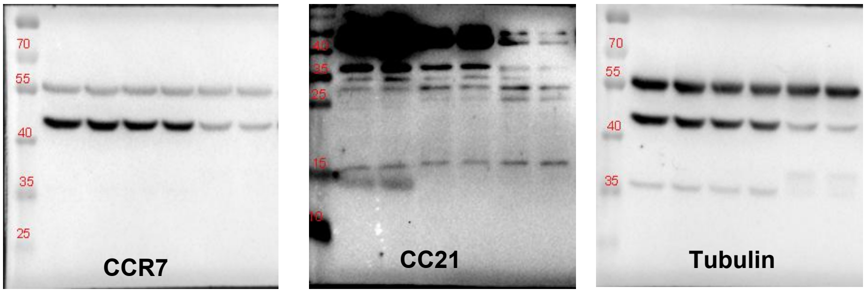

Figure 5F

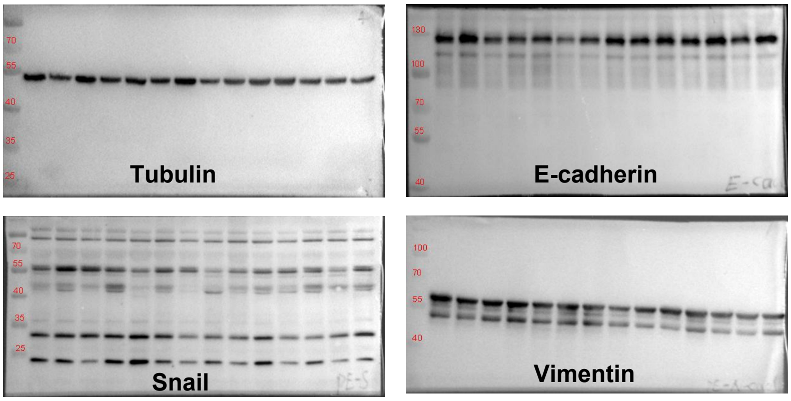

Snail	
Normal	PE
0.395969	0.287429
0.48671	0.365296
0.479341	0.386455
0.56262	0.32046
0.661809	0.338348
0.57046	0.434159

Vimentin	
Normal	PE
1.685594	1.3932
1.853756	1.407364
2.05026	1.23943
1.669381	1.129572
1.588462	1.035679
1.493618	0.82752
1.239557	0.685699

E-cadherin	
Normal	PE
1.112858	1.569234
0.908247	1.337154
1.154082	1.296404
1.10443	1.163809
0.895297	1.528334
0.836967	1.172884
	1.451145

Figure 5A

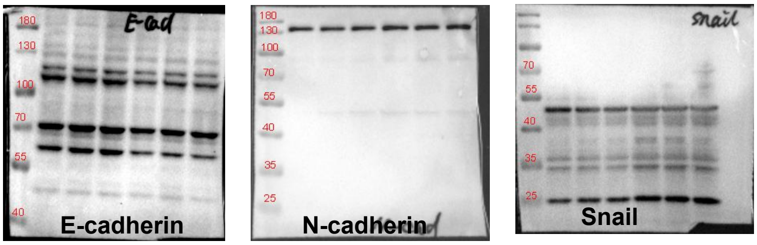

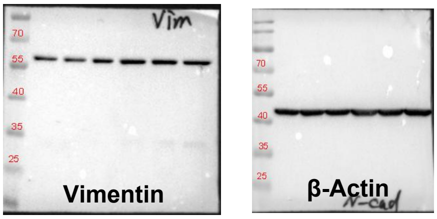

Snail	
Control	CCL21
0.284063	0.46121
0.334974	0.448747
0.313442	0.425315

E-cadherin	
Control	CCL21
0.641161	0.398338
0.675784	0.48836
0.799359	0.4492

Vimentin	
Control	CCL21
0.321548	0.7019
0.382307	0.824685
0.402928	0.581617

N-cadherin	
Control	CCL21
0.163073	0.208535
0.15084	0.197626
0.162247	0.199873

Figure 6A

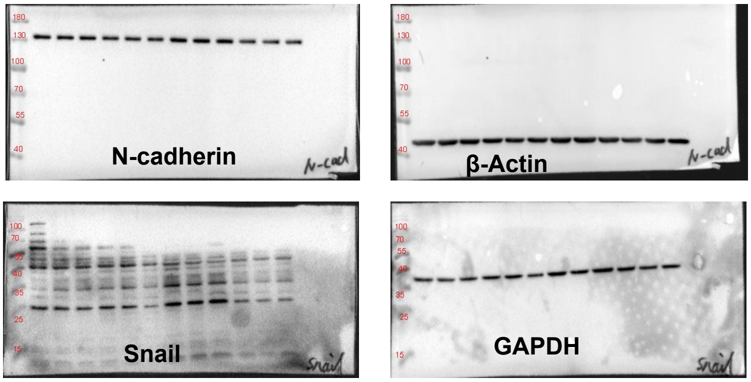

N-cadherin			
NC	CCR7i	CCL21+NC	CCL21+CCR7i
0.748822	0.603219	0.883277	0.663211
0.784198	0.656636	0.89184	0.661664
0.794111	0.696938	0.908547	0.560798

Snail			
NC	CCR7i	CCL21+NC	CCL21+CCR7i
0.701814	0.672218	1.055934	0.4191
0.731083	0.632459	0.879658	0.399393
0.594361	0.351746	0.988308	0.363198

Figure 7A

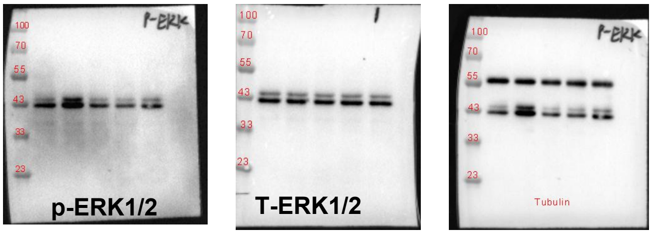

0h	2h	6h	12h	24h
0.728963	1.285584	0.708027	0.784534	0.666403
0.656526	1.339321	0.7879	0.795496	0.826366
0.656954	1.201061	0.823084	0.720609	0.743782

Figure 7C

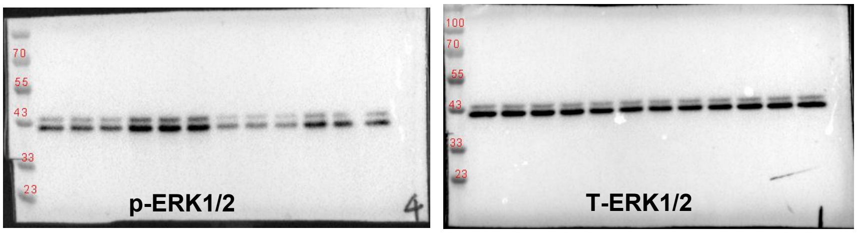

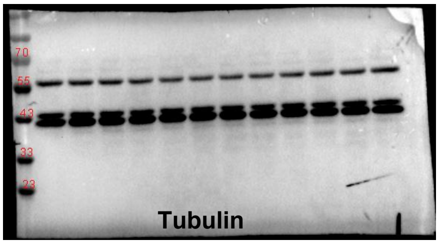

NC	CCL21+NC	CCR7i	CCL21+CCR7i
0.635807	0.98842	0.361025	0.62777
0.556311	0.977577	0.324148	0.445157
0.509132	0.983269	0.300748	0.508359

Figure 7E

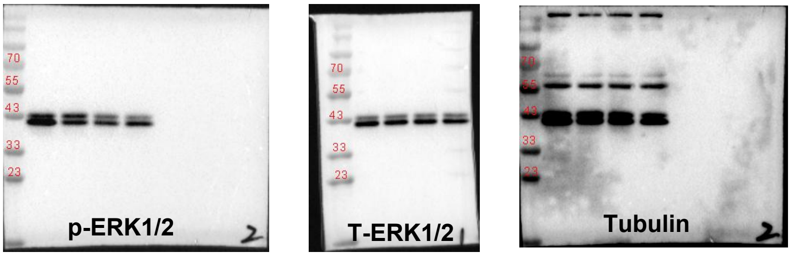

Vehicle	10nM	50nM	100nM
1.039701	0.854311	0.835369	0.785423
1.068244	0.888843	0.812899	0.755182
1.044674	0.944521	0.767445	0.783075

Figure 7G

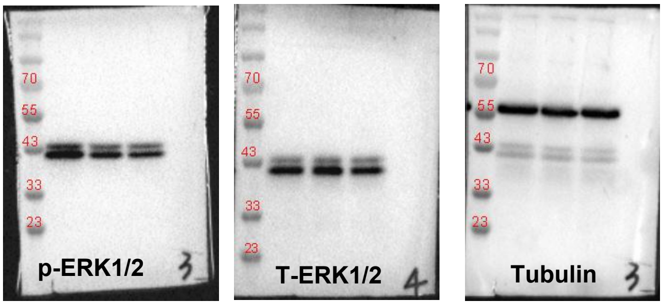

Vehicle	24h	48h
1.35605	0.95225	0.942731
1.447274	0.967477	0.918665
1.277405	0.897841	0.983769

Figure 7I

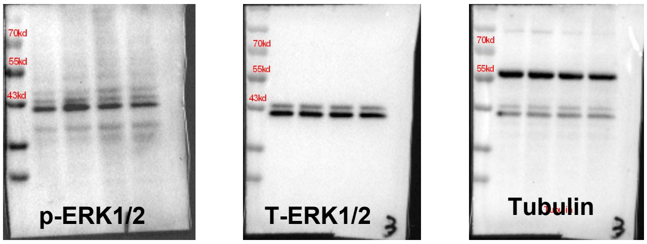

Vehicle	CCL21	ERKi	CCL21+ERKi
0.395562	0.620198	0.363412	0.451157
0.437295	0.649172	0.40804	0.385085
0.458027	0.674349	0.403899	0.559974

Figure 7K

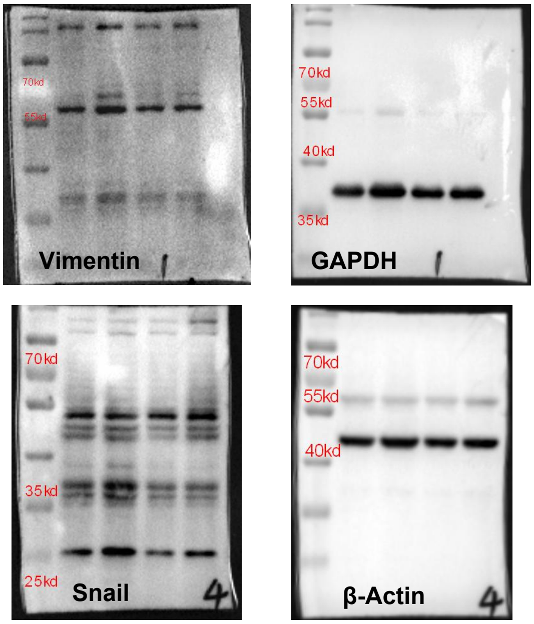

Vimentin			
Vehicle	CCL21	ERKi	CCL21+ERKi
0.13633	0.269409	0.211734	0.194684
0.157265	0.395797	0.177696	0.148229
0.157231	0.354074	0.160053	0.167217

Snail			
Vehicle	CCL21	ERKi	CCL21+ERKi
0.161554	0.381387	0.150032	0.244283
0.250928	0.530463	0.287513	0.272833
0.197854	0.4363	0.150185	0.272509

Figure 8G

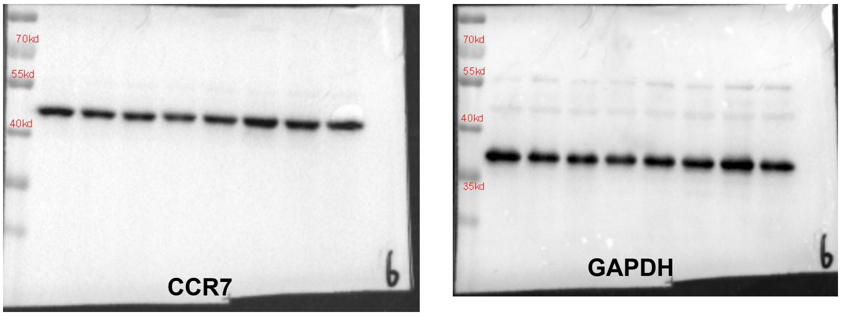

Saline	L-NAME
0.660681	0.797691
0.734414	1.08142
0.686694	0.808839
0.686626	0.878995

Figure 8I

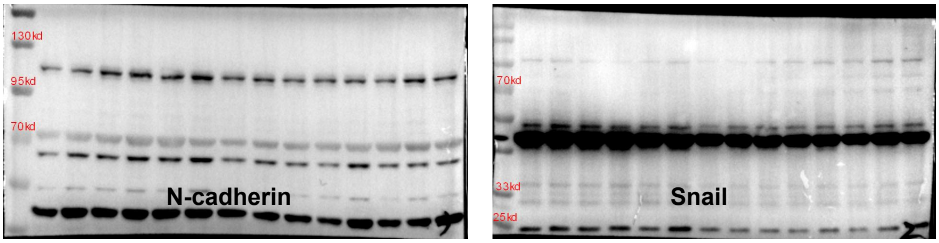

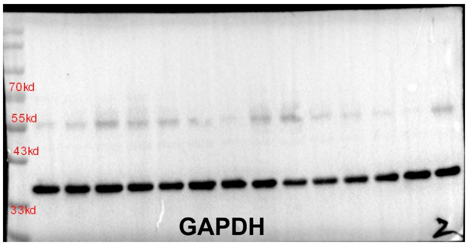

Snail	
Saline	L-NAME
0.499753	0.268486
0.517753	0.390235
0.466847	0.324383
0.564486	0.422041
0.453538	0.249948
0.619499	0.330558
0.332764	0.420454

N-cadherin	
Saline	L-NAME
0.52349	0.490132
0.41197	0.494896
0.569793	0.528697
0.699446	0.473984
0.708625	0.353188
0.717737	0.424255
0.521859	0.423438

Figure S1D

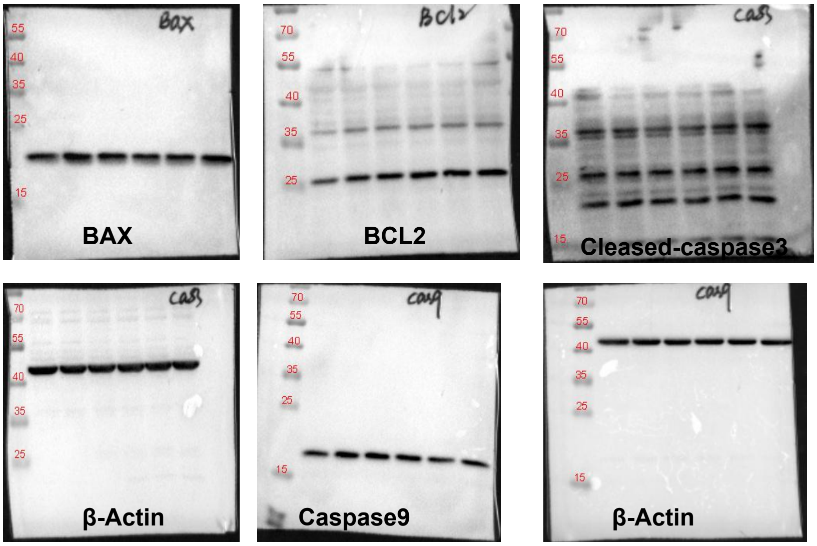

BAX	
Control	CCL21
1.045413	1.113743
1.262941	1.150391
1.331803	1.184409

BCL2	
Control	CCL21
0.363811	0.593786
0.513787	0.563593
0.561962	0.620023

Caspase9	
Control	CCL21
0.702412	0.943435
0.990565	0.710355
1.069646	0.808954

Cleased-caspase3	
Control	CCL21
0.53113	0.584092
0.68117	0.531687
0.655528	0.656139

Figure S2B

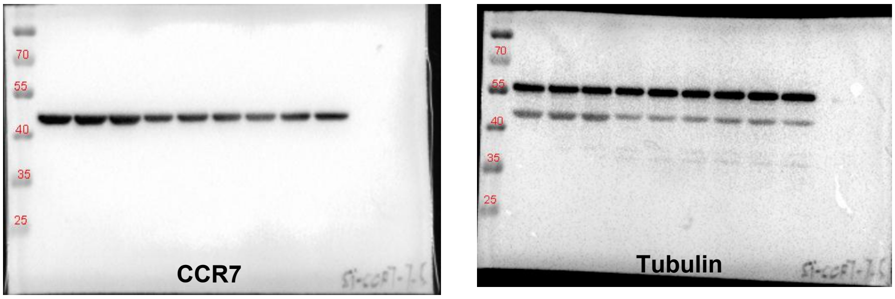

CCR7		
si-NC	si-1#	si-2#
2.772426	1.296841	0.982969
2.982288	1.275974	1.119487
2.707403	1.173446	1.289097
